# Examining the Use of an Open Digital Health Library for Professionals

**DOI:** 10.2196/resprot.3820

**Published:** 2014-11-18

**Authors:** Runar Eggen, Kjell Tjensvoll, Magne Nylenna

**Affiliations:** ^1^Norwegian Electronic Health LibraryNorwegian Knowledge Centre for the Health ServicesOsloNorway; ^2^Norwegian Knowledge Centre for the Health ServicesOsloNorway

**Keywords:** libraries, medical, access to information, information dissemination, search engine /statistics, Web log analysis

## Abstract

**Background:**

The Norwegian Electronic Health Library (The Library) is a website for health personnel. Most of the content is also open to the public. Usage statistics have risen sharply in the years 2010-2013.

**Objective:**

We wanted to find out whether the rise was caused by health personnel, the general public, or other factors.

**Methods:**

Since we lacked direct information, we had to use proxy data to shed light on our questions. We applied mixed methods (database of registered users, user survey, usage statistics, and statistics from suppliers), and triangulated between them.

**Results:**

Health personnel were our largest user group, but The Library was also accessed by students, patients, and other groups. Content in Norwegian was preferred to English language content. Concise, practical information was preferred to more comprehensive information. Patient leaflets were the most popular information type. Mobile phone visits differed from personal computer visits both in terms of time of day and what kind of information was viewed.

**Conclusions:**

The Library was used mostly by health personnel, as intended, but our data are inconclusive regarding a possible change in user groups. There was a large degree of consistency in results when using different investigation methods. The survey points toward health personnel being the largest user group, and the usage statistics show that patient leaflets are the most popular content, being viewed by both health personnel and patients.

## Introduction

The Norwegian Electronic Health Library (The Library) is a publicly funded website (Helsebiblioteket.no) for professionals, established in 2006. It is marketed to health personnel, but not to the general public. The Library provides free access to important sources of knowledge intended for health personnel, including point-of-care tools (reference works), bibliographic databases, and a large number of scientific journals [1].

The Library is also a sharing platform for guidelines, patient leaflets, and clinical procedures. It contains links to all its purchased sources, as well as open sources like rating scales, reports, summarized research, patient leaflets, and guidelines published elsewhere.

Thus, The Library is partly a traditional library service with purchased content, and partly a sharing platform where information resources are published.

Most of the content is available to anyone with a Norwegian Internet protocol (IP) address, including free access for the entire Norwegian population [2] to the five largest general medical journals and point-of-care tools like “UpToDate” and “BMJ Best Practice”. To our knowledge, The Library is the only one with national licenses for major international journals and point-of-care tools.

Users do not have to be logged in when using open access sources or subscribed material with a national access license, and may go directly to the original content provider, such as a journal’s home page. Some of the content is, for economic reasons, only available to health personnel and students. Such access is given according to recognized institutional IP addresses (hospitals, health institutions, universities, university colleges) or personal username and password assigned from The Library. As long as users are at their workplace, the IP address is recognized, so they don’t have to log in. If users are at home or anywhere else outside of the workplace they must log in to get full access to all of The Library’s resources.

From 2010 to 2013, The Library saw a sharp rise in usage. We investigated the use of The Library to find out whether this rise was caused by professionals, the general public, or other factors.

## Methods

### Overview

Since information on the individual visitors to The Library is not recorded, we had to rely on proxy variables. A proxy variable is something that is probably not in itself of great interest, but from which a variable of interest can be obtained [3].

Proxy variables serving as indicators of who is using The Library could be: where users are coming from, at what time of day the website is visited, what kind of information is most frequently used, and what kind of device people use. We collected data from several sources in order to shed light on the issue from different angles.

The data were taken from The Library’s database of registered personal users, a survey of our users within a given period, usage statistics for our website, and usage statistics from our content suppliers.

### Database of Registered Personal Users

The Library has a database of registered personal users, but we do not record their activities on the website. We have information on professional background for 48,950 of 85,270 registered users (data extracted March 31, 2014), but the quality of the data varies.

### Survey

We did a user survey from October 1 - October 9, 2013, asking the website visitors if they were health personnel, and if so, which professional group they belonged to.

The users who came to the website were served a pop-up that they could accept or reject to take part in the survey. The survey pop-up was not displayed again after the users had accepted or rejected it.

We asked the participants whether they were health personnel, students, patients/dependents, non-health personnel employees in the health services, researchers, or “other”. If they were health personnel, they were asked which personnel group they belonged to. Health personnel were also asked which sector they worked in.

### Usage Statistics

We analyzed usage statistics for The Library website from the years 2010-2013 by Google Analytics, and we looked particularly at “pageviews” of certain types of information like patient leaflets and guidelines. A “pageview” is recorded each time a user visits a webpage [4]. A single visitor can conduct many pageviews on a website. Each time the visitor returns to the page, a new pageview is recorded. Google Analytics distinguishes between new visitors and returning visitors by using cookies [5].

We analyzed bounce rates for different parts of our website. Bounce rate is defined by Google as the percentage of single-page sessions, that is, sessions in which the visitor left the site from the entrance page without interacting with the page [6].

Sessions are defined by Google as the number of individual sessions initiated by all the users of a site. If a user is inactive on the site for 30 minutes or more, any future activity is attributed to a new session. Users who leave the site and return within 30 minutes are counted as part of the original session [7].

In the analysis of pageviews for guidelines versus patient leaflets, we had data for only the first five months of 2014; therefore, we extrapolated the data as if the numbers were representative for the whole year.

### Statistics From Content Suppliers

We used available statistics from our suppliers of bibliographic databases, journals, and point-of-care tools to get data on the usage of their resources. The statistics for journals are based on successful full text downloads according to the COUNTER standard (Counting Online Usage of Networked Electronic Resources) [8]. There is a slight difference between this and a pageview, but for the practical purpose of our comparison we have chosen to ignore this. The statistics on bibliographic databases are based on number of executed searches (number of times a user has pressed the Search button) according to the COUNTER standard. The usage statistics cover the period 2010-2013, and the sources for these statistics are shown in Table 1.

The Norwegian point-of-care tool Handbook of Emergency Medicine (Legevakthåndboken) became available in September 2012. Our statistic for the Handbook of Emergency Medicine is based on Google Analytics.

In our comparison of the statistics from the suppliers with the usage statistics for our own website, we have regarded a full text download or a search as a pageview. We did this to be able to compare the data from different sources.

**Table 1:** Suppliers.

Bibliographic databases	Journals	Point-of-care-tools
OVID (AMED, Embase, Medline, Ovid Nursing, PsycINFO)	Ovid (LWW, Nursing Full Text)	UpToDate
Ebsco (Cinahl)	Informa Healthcare	BMJ (Best Practice)
	APA (PsycARTICLES)	Pharmaceutical Press (BNF Children og Martindale)
	ProQuest	Lexicomp
	BMJ (23 titles)	Gyldendal Akademiske (Handbook of Emergency Medicine, in Norwegian)
	JAMA Network (10 titles)	
	The Lancet (4 titles)	
	Annals of Internal Medicine (including ACP Journal Club)	
	New England Journal of Medicine	

## Results

### Database of Registered Users

In the database of registered personal users, we have information on professions for 56.98% (48,590/85,270) of the users (data extracted March 31, 2014). The largest group of personal users was nurses, followed by physicians, physiotherapists, psychologists, pharmacists, and assistant nurses (see Table 2).

**Table 2:** Profession and number of registered personal users of The Library (Helsebiblioteket.no).

Profession	Number of registered users n=48,590 n (%)
Nurses	12,166 (25.04)
Physicians	8924 (18.37)
Physiotherapists	2503 (5.15)
Psychologists	1806 (3.72)
Pharmacists	1070 (2.20)
Assistant nurses	804 (1.65)
Engineers	219 (0.45)

### Survey

All in all, 2563 (4.27%) visitors took part in the 9-day survey out of an estimated number of approximately 60,000 unique visitors over the same period. Not all respondents answered all the questions of the questionnaire.

A total of 55.95% (1434/2563) of the respondents reported to be health personnel, 15.02% (385/2563) students, 11.86% (304/2563) patients/dependents, 5.93% (152/2563) employees of the health services (not health personnel), 4.37% (112/2563) researchers, and 6.87% (176/2563) other.

Among the health personnel (n=1438), physicians were the largest group (38.66%, 556/1438), followed by nurses (29.62%, 426/1438), psychologists (8.34%, 120/1438), physiotherapists (5.63%, 81/1438), and pharmacists (4.66%, 67/1438).

The respondents were asked which sector of the health services they worked in: 65.51% (940/1435) of health personnel came from hospitals and specialist health services, 28.08% (403/1435) from primary care, and the rest from educational institutions, industry, research, etc.

### Usage Statistics

**Figure 1:**
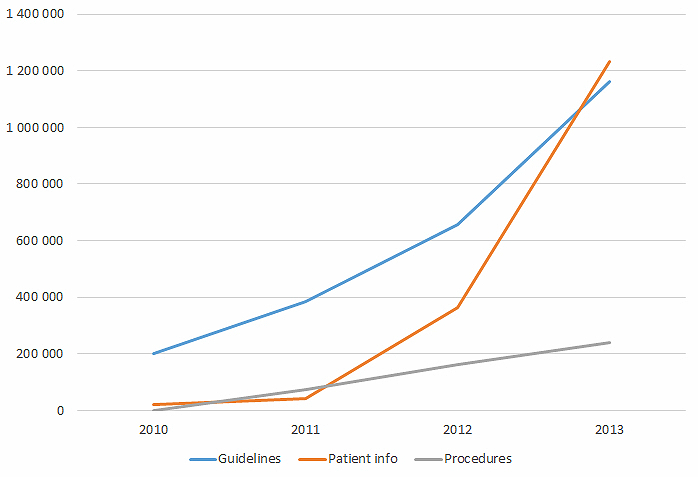
Pageviews of different information types on The Library website, Helsebiblioteket.no.

From our usage statistics, we can see that patient leaflets had the sharpest increase in pageviews from 2011 through 2013, and it was the most frequently viewed information type in 2013 (see Figure 1). There was a sharp increase in pageviews of guidelines in the period 2010-2013.

**Figure 2:**
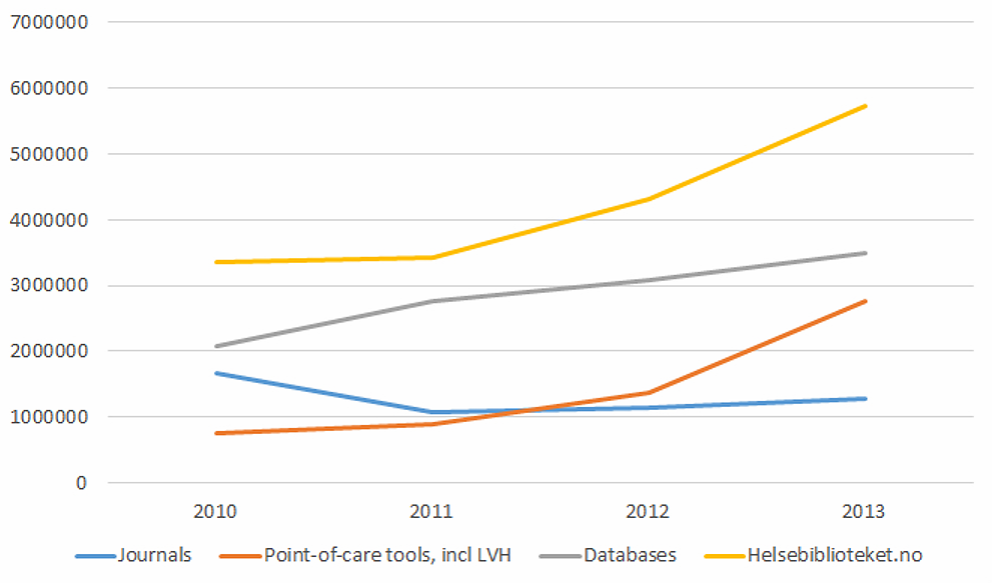
Pageviews of The Library website (Helsebiblioteket.no) versus purchased content.

The Handbook of Emergency Medicine immediately became very popular and this accounts for a large proportion of the growth in pageviews for point-of-care tools in 2012 and 2013 (see Figure 2).

The share of users coming from Google and other search engines increased throughout the period 2010-2013 and constituted approximately two-thirds of the traffic in 2013. The share of new visitors increased steadily from 2010 through 2013.

Each visitor spent a shorter time per session on the website in 2013 than in 2010.

**Figure 3:**
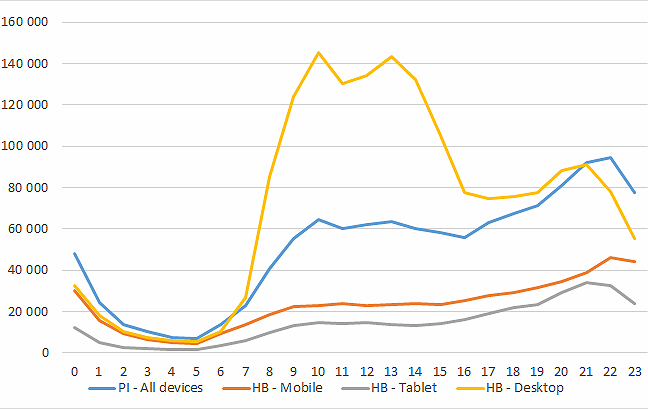
The Library (Helsebiblioteket.no) pageviews and time of day of patient information leaflets (PI) in 2013.

The share of visitors using mobile phones increased from 1.00% (10,074/1,007,395) in 2010 to 21.00% (552,859/2,632,660) in 2013. This is a general trend for websites and The Library does not differ considerably from other websites in this respect. Mobile phone users stayed for a shorter length of time on the site than personal computer (PC) users. They also viewed fewer pages than PC users. Mobile phone users spent much more time on patient leaflet pages than on guideline pages. Mobile phone users also viewed more patient leaflet pages than guideline pages. Pageviews of patient leaflets increased throughout the day, while pageviews of guidelines peaked during office hours (see Figure 3).

While visits by PC users peaked in the office hours, visits by mobile and tablet users increased steadily throughout the day (see Figure 3).

More users came from recognized workplace networks in 2013 than in 2010, but the relative share of these decreased (see Table 3).

From 2010 to 2013, we saw a 2% increase in nightly use (midnight to 8am) and a 4% increase in evening use (4pm to midnight), and a relative reduction in office hours (8am to 4pm) use.

People who viewed patient leaflets spent 4 minutes or more on each page and the bounce rate was very high.

Based on the usage statistics, the most frequently used type of information from the website was patient leaflets (see Figure 1). Other frequently used types of information on the website were guidelines and procedures.

Both the number of documents (patient leaflets, guidelines, and procedures) as well as the number of topics covered by these documents increased sharply from 2010 to 2013.

**Figure 4:**
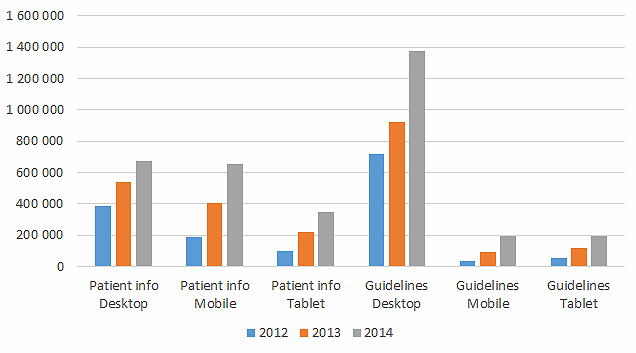
Pageviews of patient information leaflets and guidelines 2012-2014. Estimates for 2014 are based on the months January to May.

We found a difference between the kind of information that was viewed by mobile users and PC users. Patient leaflets were viewed on mobile phones almost as frequently as on PC screens. Guidelines were much more frequently viewed on PCs (see Figure 4) and very rarely on mobile devices.

**Table 3:** Usage statistics for The Library website (Helsebiblioteket.no).

Usage	2010	2011	2012	2013
Number of pageviews - all days	3,361,563	3,416,930	4,309,538	5,737,733
Number of pageviews - weekdays	2,852,482	2,903,624	3,604,040	4,724,635
Number of visits	1,007,395	1,125,686	1,605,288	2,632,660
Search engine traffic visits, n (%)	312,292 (31.00%)	427,761 (38.00%)	931,067 (58.00%)	1,676,394 (63.68%)
Direct traffic visits, n (%)	392,884 (39.00%)	382,733 (34.00%)	353,163 (22.00%)	579,185 (22.00%)
Other traffic visits, n (%)	302,219 (30.00%)	315,192 (28.00%)	321,058 (20.00%)	368,572 (14.00%)
Bounce rate	49%	55%	63%	72%
Pages per visit	3.34	3.04	2.69	2.18
Time spent per visit	4m15s	3m25s	2m38s	1m56s
New visitors	366,786 (36.41%)	453,252 (40.26%)	771,366 (48.05%)	1,308,465 (49.70%)
Mobile phones, % of visits	10,074 (1.00%)	33,771 (3.00%)	144,476 (9.00%)	552,859 (21.00%)
Tablets, % of visits	0%	0%	6%	13%
Time spent per visit, desktop	4m17s	3m36s	3m3s	2m25s
Time spent per visit, mobile phone	2m14s	2m21s	40s	40s
Pages per visit, desktop	3.33	3.05	2.90	2.61
Pages per visit, mobile phone	2.43	2.62	1.39	1.24
Visits from “workplace” networks	511,845	546,407	654,617	903,048
Office hours^b^pageviews, % of total on weekdays	1,965,360 (68.90%)	1,991,886 (68.60%)	2,378,666 (66.00%)	2,962,346 (62.70%)
Evening hours^c^pageviews, % of total on weekdays	761,613 (26.70%)	772,364 (26.60%)	1,027,151 (28.50%)	1,450,463 (30.70%)
Overnight hours^a^pageviews, % of total on weekdays	125,509 (4.40%)	142,278 (4.90%)	198,222 (5.50%)	311,826 (6.60%)

^a^overnight hours: midnight to 8am

^b^evening hours: 4pm to midnight

^c^office hours: 8am to 4pm

### Statistics From Content Suppliers

Based on statistics from our suppliers (Table 1), the usage of journals, point-of-care tools, and bibliographic databases showed a steady growth in the period we analyzed (2010-2013).

The statistics from our suppliers combined with the usage statistics (see Figure 2) make it possible to compare pageviews of journals, point-of-care tools, database searches, and The Library website. Pageviews in the website increased more than the pageviews of purchased sources. In 2013, there were 5.7 million pageviews of the website pages, against 4.0 million pageviews for purchased resources like point-of-care tools and journals. Point-of-care tools rose sharply from September 2012 when the Norwegian language point-of-care tool Handbook of Emergency Medicine was introduced. The Handbook of Emergency Medicine is available online to anyone with a Norwegian IP. It can also be downloaded as an app. This source now has more pageviews than all other point-of-care tools combined.

## Discussion

### Principal Results

The Library website and its purchased content are widely used by both health personnel and the general public. Over the last few years, there has been a sharper rise in the use of the website than in the use of most content located outside the website. This could be due to better visibility in Google and other search engines, but that is probably not the only reason. There has also been an increase in the number of documents published on the website.

Norwegian language seems to play a role. Norwegian guidelines, patient leaflets, and the Handbook of Emergency Medicine had a sharper rise in popularity than the rest of our content. In fact, the Handbook of Emergency Medicine had more pageviews than all the other point-of-care tools combined in 2013. A study among Norwegian physicians has shown that it is highly valued to get information in their mother tongue [9].

Cultural factors, ease-of-use, and how practical the information is, may also play a role. The Handbook of Emergency Medicine is very concise and practical, but it covers only a fraction of the topics that our English language point-of-care tools cover. Patient leaflets are also very concise and highly popular among our users. A study on preferred sources of information for primary care physicians from 1997 states that “Information resources for answering clinical questions should be readily available, familiar, and quick to use” [10].

There was large growth in the number of mobile phone users visiting the website. We found that visits from PC users peaked during office hours, while visits from mobile users increased throughout the day. Nicholas et al [11] and Cronk [12] have shown that there is a difference in the time of day when PC users visit a website and when mobile users visit the same site, and the difference is the same as we found: PCs peak in office hours and mobile phones peak in the evening. Even so, we still have more PC users in the evening than we have mobile users.

Nicholas et al [11] found that mobile users typically viewed other kinds of material than PC users, typically shorter texts and fewer pages. We found the same—mobile phone users viewed relatively more patient leaflets and fewer guidelines than PC users on The Library website.

### Strengths and Limitations

The use of different methods to analyze the usage is a strength of this study. It is also a strength that we have followed the usage over several years.

There are some weaknesses regarding the database, survey, and the Google Analytics data.

The data quality of the database of registered personal users is not optimal. The users report their professional position in free text, which makes classification difficult. Our survey covered only 9 days, and the response rate was low. According to the survey software supplier Surveygizmo [13], external surveys like customer satisfaction surveys generally get 10-15% response rates, while internal surveys get 30-40% response rates. Our survey had an estimated response rate of 4%. This estimate is probably too low, since we didn’t measure non-responders directly, but based the estimate on Google Analytics’ unique visitor statistic and cookies.

Google Analytics is based on samples of data and hence may be skewed. We have, however, followed the use of the website through Google Analytics over 4 years, and the tendencies are consistent over all these years.

Google Analytics’ new/returning visitor statistic may be unreliable since it uses cookies. Cookies are specific for each Web browser and device, so the same person will be counted as a new visitor if he or she first visits a site from the office PC, then from the home PC, and again if he or she changes Web browser, deletes cookies, or visits the same site from a mobile phone. The reported ratio of new users is therefore probably exaggerated [5]. The same applies to statistics of unique visitors.

Some of the Google Analytics data give a skewed picture even if they are technically correct. Guidelines pageviews, for example, are reported a bit too high, due to each guideline being split into several documents on our website.

### Interpretation

The data from the usage statistics for the website indicate that patient leaflets are becoming more popular. At the same time, the survey data indicate that the majority of users are health personnel. How can this be explained?

The Library is primarily a link portal. Patient leaflets are, along with guidelines, recommendations, and procedures, the only content that is published on the website itself. The same development as we have seen for patient leaflets can be seen for guidelines. Their use (registered as pageviews) also increases sharply. In other words, what we are seeing is an increase in the usage of content that is located on the website itself.

The percentage of users coming from the Google search engine underpins this. The content of the website is more visible to Google than the content of our purchased material. The proportion of users coming from search engines has increased from 31% to 64%.

The proportion of new visitors increased from 36% to 50% over three years. If this is real change and not just an artefact due to the use of cookies, this could very well be a sign that the search engine optimization is starting to pay off. The Library website, Helsebiblioteket, has a Google PageRank of 7/10, which is regarded as very high (on par with the newspaper The Guardian). Google PageRank is an algorithm used by Google Search to rank websites in their search engine results. PageRank is a way of measuring the importance of website pages [14] and is given as a number between 0 and 10 [15].

The high Google ranking may lead more people from outside the ranks of health personnel to our pages. This may be one of the reasons why time spent on the portal and pages per visit both go down. Some of these visitors probably don’t find what they expect on the website.

Is it patients then who read the patient leaflets? Our data give us an indication, but are not conclusive.

The hour of the day of viewing of patient leaflets shows a pattern that resembles both the visits by PC users and the visits by mobile phone users. There is a small peak in office hours, but the number of pageviews increases again from 4 pm and peaks at 10 pm. This indicates that a large proportion of the readers of patient leaflets are the general public or patients, or at least not people at work. But the peak in office hours indicates that the patient leaflets are also used by health personnel at work.

We have to remember that most health personnel are not physicians, and they might very well make good use of patient leaflets. It may also be that physicians visit the website and print the patient leaflets for their patients. Professionals and patients alike seem to embrace simple, short, and trustworthy information.

The inclusion of the patient in the decision process is becoming more common in health care. To make this inclusion meaningful, the patients need to be informed. According to Longo [16], patients have different information needs depending on where they are in the process. Since The Library is quite unique with its national licenses for highly respected point-of-care tools and journals, we didn’t find any other studies examining patients’ use of similar websites.

The importance of visibility in search engines for medical websites was shown by Giustini as early as 2005 [17]. This visibility also makes it possible to retrieve professional information using devices like mobile phones far away from the office.

We found two studies on how a large public website is accessed with different devices. Cronk studied the use of the British government website gov.uk and found that mobile phones and tablets were used more in the evenings and weekends, while desktop/laptop PCs dominated the office hours [12]. Nicholas et al compared mobile users with PC users of Europeana, the European fulltext cultural website. They found that mobile users were the fastest growing group and that their visits were different from PC user visits. Mobile visits were typically shorter, less interactive, and less content was viewed per visit. Mobile use peaked in the evenings and weekends. They found that tablet users behaved very much like PC users [11].

We found the same pattern as Cronk [12] and Nicholas et al [11]. PCs peaked during working hours, while mobile phones increased throughout the day and peaked around 10 pm. We also found that pageviews of patient leaflets followed a pattern in between the PCs and mobile phones. Patient leaflets showed a small peak during office hours and a larger peak in the evening. This indicates that patient leaflets are actually read by the general public, but they are probably also read or handed out by health personnel during office hours.

We are now redesigning The Library website, partly to make it more mobile friendly, and the findings in this study are being taken into consideration in this process. It was decided to keep the patient information leaflets, partly as a consequence of the findings in this study.

### Conclusion

We assume our largest user group is health personnel and students, as intended. The Library is widely used among health personnel, and to some extent also by the public. Norwegian language content is more popular than English language content. We cannot conclude whether the high and increasing popularity of patient leaflets is caused by patients using The Library more than before.

## Abbreviations

COUNTER: Counting Online Usage of Networked Electronic Resources
IP: Internet protocol
PC: personal computer
